# The impact of genetic heterogeneity on biomarker development in kidney cancer assessed by multiregional sampling

**DOI:** 10.1002/cam4.293

**Published:** 2014-08-14

**Authors:** Alexander Sankin, Abraham A Hakimi, Nina Mikkilineni, Irina Ostrovnaya, Mikhail T Silk, Yupu Liang, Roy Mano, Michael Chevinsky, Robert J Motzer, Stephen B Solomon, Emily H Cheng, Jeremy C Durack, Jonathan A Coleman, Paul Russo, James J Hsieh

**Affiliations:** 1 Urology Service, Department of Surgery, Memorial Sloan-Kettering Cancer CenterNew York City, New York; 2 Human Oncology and Pathogenesis Program, Memorial Sloan-Kettering Cancer CenterNew York City, New York; 3 Department of Epidemiology and Biostatistics, Memorial Sloan-Kettering Cancer CenterNew York City, New York; 4 Interventional Radiology Service, Department of Radiology, Memorial Sloan-Kettering Cancer CenterNew York City, New York; 5 Genitourinary Oncology Service, Department of Medicine, Memorial Sloan-Kettering Cancer CenterNew York City, New York; 6 Department of Pathology, Memorial Sloan-Kettering Cancer CenterNew York City, New York; 7 Department of Medicine, Weill Cornell Graduate School of Medical SciencesNew York City, New York

**Keywords:** Biomarker, genetic heterogeneity, kidney cancer, renal biopsy, renal cell carcinoma

## Abstract

Primary clear cell renal cell carcinoma (ccRCC) genetic heterogeneity may lead to an underestimation of the mutational burden detected from a single site evaluation. We sought to characterize the extent of clonal branching involving key tumor suppressor mutations in primary ccRCC and determine if genetic heterogeneity could limit the mutation profiling from a single region assessment. Ex vivo core needle biopsies were obtained from three to five different regions of resected renal tumors at a single institution from 2012 to 2013. DNA was extracted and targeted sequencing was performed on five genes associated with ccRCC (von-Hippel Lindau [*VHL*], *PBRM1*, *SETD2*, *BAP1*, and *KDM5C*). We constructed phylogenetic trees by inferring clonal evolution based on the mutations present within each core and estimated the predictive power of detecting a mutation for each successive tumor region sampled. We obtained 47 ex vivo biopsy cores from 14 primary ccRCC's (median tumor size 4.5 cm, IQR 4.0–5.9 cm). Branching patterns of various complexities were observed in tumors with three or more mutations. A VHL mutation was detected in nine tumors (64%), each time being present ubiquitously throughout the tumor. Other genes had various degrees of regional mutational variation. Based on the mutations' prevalence we estimated that three different tumor regions should be sampled to detect mutations in *PBRM1*, *SETD2*, *BAP1*, and/or *KDM5C* with 90% certainty. The mutational burden of renal tumors varies by region sampled. Single site assessment of key tumor suppressor mutations in primary ccRCC may not adequately capture the genetic predictors of tumor behavior.

## Introduction

The incidence of renal cell carcinoma (RCC) continues to rise in the United States among all racial and ethnic groups. There are ∼65,000 new cases and over 13,000 deaths per year due to kidney cancer [1]. RCC encompasses a family of tumors, each with distinct histology, variable metastatic potential, and genetic landscape resulting in diverse growth kinetics and responses to treatment [2]. The most common RCC subtype is clear cell (ccRCC), which accounts for 54% of all renal cortical tumors but 90% of those that ultimately metastasize [3].

Although molecular mechanisms involved in the pathogenesis of ccRCC have recently been identified leading to the development of targeted therapy with improved survival compared to cytokines and chemotherapeutic agents, eventually tumors develop resistance leading to disease progression [4]. Additionally, there are highly variable interpatient responses to systemic therapy, which has lead to growing interest in personalized treatment regimens based on the molecular tumor profiles.

A substantial obstacle to appropriate selection of precision therapies is intratumor genetic heterogeneity. Genomic studies in breast, liver, pancreas, and kidney tumors have revealed regionally diverse mutational landscapes in primary tumors as well as metastatic sites and sites of local recurrence after treatment [5–8]. The notion that a tumor's genetic landscape has regional variability may help to expose the etiologies of treatment failure and drug resistance [9]. Personalized medicine strategies typically rely on a single site evaluation to direct therapy [10]. A single site assessment, however, may underestimate the mutational burden within a tumor and thus, presents an obstacle for selecting the appropriate treatment options. This also presents a challenge for the validation of biomarkers using single region molecular profiling [11].

Tumor initiation in ccRCC is thought to be due to functional loss of chromosome 3p [12]. Although the most frequently altered gene in this locus is von-Hippel Lindau tumor suppressor (*VHL*), there does not appear to be any association with mutation status to tumor aggressiveness and clinical outcome, making *VHL* a poor prognostic biomarker. Other tumor suppressors involved in epigenetic regulation have been implicated in ccRCC tumorigenesis, as evidenced by three large-scale multiplatform sequencing studies [13–15]. These studies confirmed recurrent mutations in the chromatin-modulating genes polybromo 1 (*PBRM1*) [16], SET domain containing 2 (*SETD2*) [17, 18], BRCA1-associated protein-1 (*BAP1*) [19], and lysine (K)-specific demethylase 5C (*KDM5C*) [17] in kidney cancer. We and others have reported the associations of *PBRM1*, *SETD2*, *BAP1*, and *KDM5C* mutations with advanced stage, grade, and tumor invasiveness [20, 21], and discovered that *SETD2* and *BAP1* mutations are associated with lower cancer-specific survival rates [21, 22]. The strong linkage between these chromatin-modulating genes and tumor behavior, along with the increasing importance of epigenetic regulation in cancer, suggest these genes may be valuable biomarkers and detection of their mutation status may aid in clinical decision making [23–25].

In this study, next-generation DNA sequencing was performed on multiple tumor regions in primary ccRCC's, targeting coding regions of key tumor suppressor genes with known associations to ccRCC. From these data, evolutionary phylogenetic trees were constructed and the predictive power of additional sampling taken in a single renal tumor was inferred using statistical models.

## Methods

### Clinical samples

Ex vivo 18 and 20 gauge core needle biopsies 2 cm in length were obtained from three to five geographically different, nonnecrotic, regions of primary renal tumors to capture the known high-genomics heterogeneity of ccRCC that were resected via partial or radical nephrectomy at a single institution from October 2012 to June 2013. All patients had signed informed consents for tissue utilization, and our institutional review board had approved the study. DNA was extracted for next-generation sequencing.

### Sequencing and mutation analysis

Targeted sequencing of the coding regions for five genes with known associations to RCC (*VHL*, *PBRM1*, *SETD2*, *BAP1*, and *KDM5C*) was performed using a Miseq desktop sequencer (Illumina, San Diego, CA). Base Space (https://basespace.illumina.com/home/index) was used for quality control, trimming, and mapping. The median coverage across all samples was 950×, with 98% of targeted regions covered >100×. An adopted GATK mutation calling pipeline with the following computational steps was applied: (1) marking duplicates (2) local realignment around indels (a list of known indel sites from Miller data set and 1000 Genome were used as targets for realignment) (3) base quality score recalibration through known sites from Miller data, 1000 Genome, and dbSNP (4) variant calling through HaplotypeCaller and UnifiedGenotyper.

All missense, nonsense, and indel mutations were included in our analysis if present in at least 5% of reads with a minimum sequencing coverage of 10. Single base pair mutations were excluded from analysis if present in germline SNP database (dbSNP). Additionally, all synonymous mutations were excluded from final analysis.

All traces were manually reviewed by A. S., using Integrative Genomics Viewer (Broad Institute of Massachusetts Institute of Technology and Harvard, Cambridge, MA). All missense variants detected in less than all cores within a tumor or detected with a coverage <10× were validated by repeat polymerase chain reaction (PCR) amplification and Sanger resequencing of unamplified diagnostic DNA with the exception of known VHL point mutations from the COSMIC database or VHL frameshift mutations. Furthermore, among all the mutation reads, all are far above five reads of the same mutations within individual tumors, except the BAP1 L709F missense mutation at four reads.

### Phylogenetic tree construction

Mutations were defined as “shared” or “nonshared” based on the number of cores they were present in within a single tumor. A shared mutation is a mutation detected in all cores within a single tumor. A nonshared mutation is a mutation absent in at least one core. Phylogenetic trees were constructed by inferring clonal evolution. This was done by fitting the observed mutations present within each core into a single tree for each tumor.

### Estimating predictive power of each biopsy

In order to calculate the predictive power of each subsequent biopsy, the intratumor mutation prevalence, defined as the proportion of tumor cells with a mutation, was estimated for each gene. This was accomplished by calculating the proportion of all biopsies with a mutation in that gene across all patients who have at least one biopsy with the mutation. The underlying assumptions for this estimate are that (1) biopsies are independent and randomly distributed within the tumor and (2) patients without the detected mutation in at least one of the biopsies do not have that mutation. We then used binomial distribution and the estimated intratumor mutation prevalence for each gene to calculate probability of detecting a mutation in that gene as a function of total number of biopsies. We focused our analysis on genes *PBRM1*, *SETD2*, *BAP1*, and *KDM5C* because of their known association with ccRCC prognosis.

## Results

### Mutation frequencies

We obtained a total of 47 ex vivo biopsy cores on primary renal tumors from 14 patients with ccRCC. Table 1 lists the clinicopathologic characteristics of all tumors. The median patient age was 52.5 (IQR 46.0–57.8) and the median tumor size was 4.5 cm (IQR 4.0–5.9 cm). The median follow-up on our cohort was 7.6 months. Due to our relatively short follow-up time, we chose not to extrapolate any conclusions on patient outcomes and heterogeneity.

**Table 1:** Clinicopathologic characteristics of patients with renal tumors.

Patients	*N* = 14
Median age (IQR)	52.5 (46.0–57.8)
Median tumor size (cm) (IQR)	4.5 (4.0–5.9)
Histology (%)	
Clear cell	14 (100%)
Grade (%)	
1–2	5/14 (36%)
3–4	9/14 (64%)
Stage (%)	
I–II	8/14 (57%)
III–IV	6/14 (43%)
Neoadjuvant Tx	0/14 (0%)

**Figure 1:**
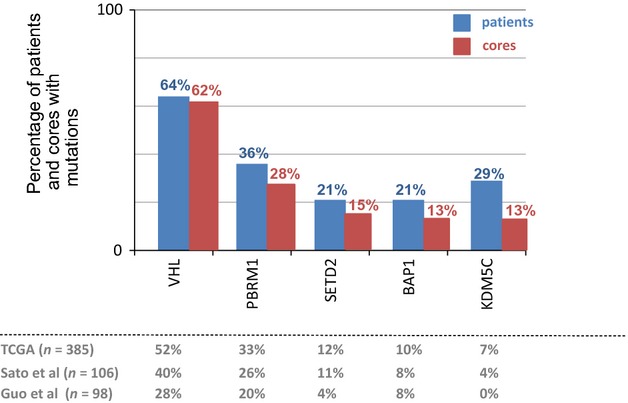
Overall mutation rate by patient (in blue, *n* = 14) and by core (in red, *n* = 47). Comparison cohort frequencies are shown in gray.

The overall mutation rates for each studied gene are represented in Figure 1. The most prevalent gene harboring a mutation was *VHL*, which was present in 9/14 patients (64%). The chromatin modulators *PBRM1*, *SETD2*, *BAP1,* and *KDM5C* were present in five (36%), three (21%), three (21%), and four (29%) patients, respectively. When compared to mutation frequencies identified in three other large-scale sequencing studies [13–15], we detected a greater mutational burden across all genes.

A similar rate of *VHL* mutations was observed when we assessed the mutational burden of each individual core, revealing mutations in 29/47 cores (62%). The mutation rates of the chromatin modulator genes decreased when assessed by each core taken. Specifically, there was a *PBRM1* mutation detected in 13/47 cores (28%), a *SETD2* mutation in 7/47 cores (15%), a *BAP1* mutation in 6/47 cores (13%), and a *KDM5C* mutation in 6/47 cores (13%).

**Figure 2:**
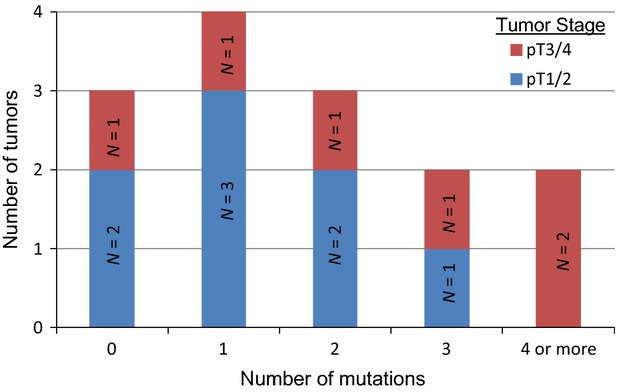
Number of mutations per tumor.

Figure 2 illustrates the number of unique mutations observed in each tumor. Three tumors had no detectable mutations, four tumors had only one mutation, and the remainder of tumors had two or more mutations. Of note, both tumors harboring four or more mutations were advanced stage.

### Genetic branching

We observed intratumor genetic branching patterns of multiple complexities, ranging from a single shared mutation to a shared mutation and multiple nonshared mutations within a tumor.

**Figure 3:**
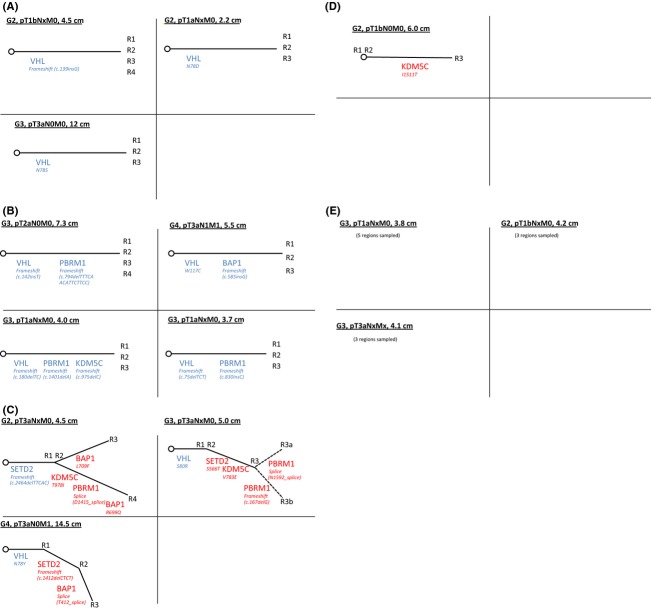
(A) Phylogenetic trees of tumors with a single shared mutation (grade, stage, and size notated in black, shared mutations in blue, nonshared mutations in red)(R1–R4 designate each biopsy core). (B) Phylogenetic trees of tumors with multiple shared mutations. (C) Phylogenetic trees of tumors with shared and nonshared mutations (R3a and R3b represent distinct regions of one biopsy core in which different mutations were detected in a single gene). (D) Phylogenetic tree of tumor with nonshared mutations only. (E) Tumors with no mutations.

Figure 3A depicts the phylogenetic trees of tumors containing a single shared mutation only. Of these three tumors, each of them contained a single mutation in the *VHL* coding region.

There were four tumors containing at least two shared mutations (Fig. 3B). Amongst this group, *VHL* was the most commonly mutated gene (4/4 tumors), followed by *PBRM1* (3/4 tumors). This is consistent with previous findings that *PBRM1* mutations likely represent the second genetic event in tumor initiation after loss of *VHL* [22].

There were three tumors containing both shared and nonshared mutations (Fig. 3C). The regional mutational diversity was unique in each tumor. These three tumors had the highest degree of genetic complexity among the whole cohort and of note were all pathologic stage T3a. The tumor with the most mutated genetic landscape contained a shared *SETD2* mutation and four nonshared mutations (in genes *KDM5C*, *BAP1*, and *PBRM1*). This tumor also contained two distinct mutations in *BAP1*, each detected in different cores.

Another branching pattern observed was one of nonshared mutations only (Fig. 3D). There was a single tumor containing a nonshared *KDM5C* mutation.

Last, there were three tumors with no mutations detected (Fig. 3E).

### Heterogeneity of mutations

**Figure 4:**
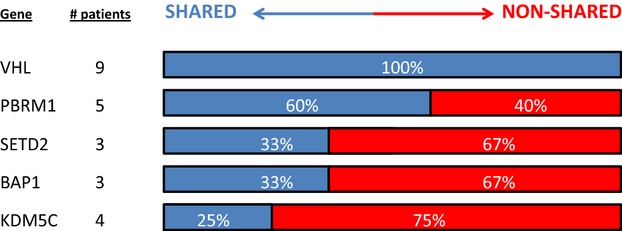
Variability of mutation patterns.

The five genes sequenced exhibited unique patterns of mutation prevalence. As shown in Figure 4, some genes had a propensity to be mutated in all cores while others tended to be absent in at least one core. For example, when *VHL* mutations were present, they were identical and ubiquitous in all core samples, likely reflecting an early event in tumorigenesis. In contrast, when *PBRM1*, *SETD2, BAP1,* or *KDM5C* mutations were present, they were present in all cores 60%, 33%, 33%, and 25% of the time, respectively.

### Predictive power of each subsequent biopsy

**Figure 5:**
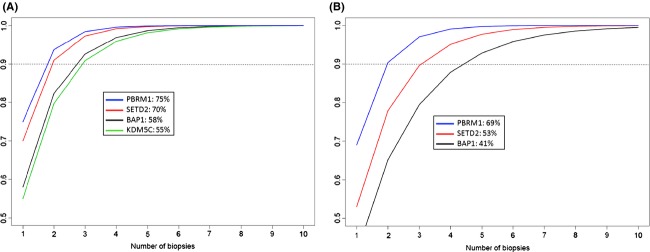
(A) Probability to detect a mutation with each successive biopsy (Legend shows gene name: % of tumor volume containing mutation when present). (B) Probability to detect a mutation with each successive biopsy based on the estimates from data in Gerlinger et al. independent cohort (Legend shows gene name: % of tumor volume containing mutation when present).

Figure 5A shows the probability of detecting a prognostic gene mutation based on each successive biopsy taken. Based on our cohort (*n* = 14), we estimate that among patients harboring a mutation in *PBRM1*, *SETD2*, *BAP1*, or *KDM5C*, the mutation would be present in 75%, 70%, 58%, and 55% of the tumor, respectively. Thus, based on the binomial calculation, in order to detect a mutation in *PBRM1*, *SETD2*, *BAP1*, and/or *KDM5C* with 90% certainty, a tumor would need to be genetically profiled in at least three locations.

Similar plots demonstrating prediction power for successive sampling sites were constructed using estimated mutation prevalences from a sequencing study by Gerlinger et al. [26]. In this study, the authors performed whole-exome sequencing on up to 10 regions in primary ccRCC's. These data provided us with an independent cohort to compare with our findings. As shown in Figure 5B, the estimated mutation prevalences for *BAP1*, *SETD2*, and *PBRM1* mutations were 41%, 53%, and 69%, respectively, compared to our cohort which was 58%, 70%, and 75%, respectively. Of note, we did not include *KDM5C* in the supplementary analysis since this mutation was only detected in one tumor in the independent cohort and had a near ubiquitous prevalence in that tumor. As expected, the lower estimated mutation prevalence in this 10 patient cohort yielded a slightly lower prediction power per sampling site. For example, a minimum of three samples would be necessary to detect a *SETD2* or *PBRM1* mutation with 90% certainty, but five samples would be necessary to detect a *BAP1* mutation.

## Discussion

In this study, we investigated the intratumor mutational heterogeneity present in the most common genes associated with ccRCC. The geographic distribution of shared and nonshared mutations obtained from core needle biopsies enabled us to derive common ancestry and clonal evolution within a primary tumor. We illustrated these evolutionary changes with phylogenetic trees, which ranged from simple and nonbranching to complex trees with multiple branches. We then calculated the minimum number of tumor regions necessary to sample when searching for critical gene mutations.

This method of interpretation through regional sequencing has been previously utilized in a report by Gerlinger et al. in which they characterized the complexity of clonal branching in four patients with metastatic ccRCC [6]. Through whole-exome sequencing and chromosome aberration analysis of multiple spatially distinct sites within primary tumors and metastatic lesions, the authors elegantly demonstrated intratumor genetic heterogeneity and constructed phylogenetic trees. In a subsequent study by Martinez et al. [27], the authors collected multiple spatially distinct ex vivo biopsies from advanced stage ccRCC's and analyzed copy number alterations between sites. They observed a high degree of intratumor variability with respect to DNA copy number alterations. Similarly, Voss et al. [28] have demonstrated both shared and nonshared somatic mutations and copy number alterations among different tumor regions in primary and metastatic ccRCC. Furthermore, these tumors exhibited convergent mutation evolution in common molecular pathways which may explain their exceptional response to systemic therapy targeted to that pathway. An important distinction between our study and these previous reports is that we analyzed tumors of all stages and did not limit sequencing to late-stage tumors only.

We detected an overall increased mutation frequency rate among all genes when compared to other large-scale sequencing studies [13–15], although this observation was most prominent in genes with nonubiquitous mutations. For example, *BAP1* and *KDM5C* were mutated in 21% and 29% of tumors in our cohort, respectively, compared to 10% and 7% in the TCGA cohort [13], 8% and 4% in the Sato et al. cohort [14], and 8% and 0% in the Guo et al. cohort [15]. Of note, the mutation rate of these genes per biopsy core (rather than per tumor) is more similar to the above mentioned cohort mutation rates (13% and 13%, respectively). This phenomenon suggests that true mutation rates may be higher than previously reported, particularly in genes that may become aberrant later in tumor evolution.

Our data demonstrate that prognostic gene mutations can be detected using core needle biopsy samples in one region of a tumor that are not detected in adjacent regions. The predictive power of detecting a prognostic gene mutation increases with each successive sampling site within a primary renal tumor. In our cohort, we estimated that a minimum of three tumor regions must be sampled to detect mutations in *PBRM1*, *SETD2*, *BAP1*, and/or *KDM5C* with 90% certainty. The detection probability reached a plateau after four to five samples.

In contrast to our results, the Gerlinger et al. study [26] observed saturation in detecting mutation events in only three out of 10 patients. Through whole-exome sequencing, they were able to capture a much larger number of unique mutations for each additional biopsy taken, but one must keep in mind that most of these mutations have unknown prognostic value and likely act as passengers, rather than drivers. We focused our analysis on genes with known prognostic value, thus eliminating the background noise of passenger gene mutations. The lack of saturation observed in the majority of the Gerlinger et al. cohort is likely a consequence of nonsignificant mutation events.

Similar results were obtained when we applied our statistical model to the regional mutation data on this independent study of 10 primary renal tumors [26]. These findings are consistent with our conclusion that three spatially distinct samples must be obtained to detect a SETD2 and/or PBRM1 mutation with 90% certainty. The independent cohort demonstrated a slightly lower mutation prevalence for BAP1, however, resulting in a detection certainty of 80% with three samples.

There are two fundamental differences between our and the Gerlinger studies. First, we chose to sample three to five cores to specifically address the daily issues that urological and medical oncologists face in terms of primary tumor heterogeneity. The clear distinction between our and the Gerlinger studies is that only two out of 14 patients presented with metastatic disease in our cohort, whereas the majority presented with metastatic disease in the Gerlinger studies (4/4 metastatic in [6], 8/10 metastatic in [26]). Future studies with larger cohorts will be required to confirm our findings. Second, we chose a specific five-gene set instead of whole exomes since our pilot study focuses on specific genes and their associated risks, whereas the Gerlinger studies primarily focuses on tumor evolution.

An important implication of this study is that we must question the accuracy of a single site molecular assessment in characterizing the overall genetic landscape for renal tumors. Single site evaluation to detect prognostic gene mutations, even with multiple samples obtained at that site, may lead to substantial false-negative marker assessments; this represents an impediment to clinically relevant tissue sampling. The presence or absence of a prognostic gene mutation may impact the decision to perform surgical resection or ablative therapy for a small renal mass rather than observation. Additionally, as we enter a new era of molecular pathway-based therapeutics, detection of gene mutations not only plays a role in patient risk stratification, but also in selection of the appropriate targeted therapies.

One limitation of our study is that we did not correlate the genetic data with biopsy histology, although we felt that this more closely resembled the real world clinical scenarios in which a pathologist would not evaluate each individual core. Additionally, certain assumptions were made in order to estimate the predictive ability of each biopsy, including that each core was taken independently and at a random tumor location, and that if a mutation was not detected then it was assumed that it did not exist elsewhere in the tumor.

The clinical implications of these results are yet to be determined. One must consider that our biopsies were captured from the nephrectomized specimens, enabling us to easily sample different tumor regions. This methodology is not yet applicable for in situ cases without increasing risk of bleeding and adjacent organ injury along with prolonging anesthesia time. Multi-site tumor sampling after surgical resection, however, should be considered for stratifying postnephrectomy cancer-specific risk and for selection of subsequent systemic therapies. It may also be a worthwhile strategy to combine DNA from three to four tumor regions and sequence as a single specimen. This process would maximize mutation detection yield while minimizing the cost of sample sequencing.

## Conclusions

The mutational burden of renal tumors varies by region sampled. Ex vivo core needle biopsies reveal regional variations in key tumor suppressor mutations associated with ccRCC. Single site assessment of prognostic genetic mutations is unlikely to reflect the heterogeneous nature of kidney cancer. This presents a challenge when attempting to capture genetic predictors of tumor behavior by sampling a single site.
